# Supplementary material for: Reference flow: reducing reference bias using multiple population genomes
Source: Genome Biol. 2021 Jan 4;22:8. doi: 10.1186/s13059-020-02229-3 (PMC7780692; doi:10.1186/s13059-020-02229-3)
Supplement: Supplementary file 1 — Additional file 1 Supplementary information. Contains two Notes S1–S2, Tables S1–S4, and Figures S1–S17. [file 13059_2020_2229_MOESM1_ESM.pdf]

Supplement for: Reference flow: reducing reference bias using multiple population genomes

Nae-Chyun Chen[1], Brad Solomon[1], Taher Mun[1], Sheila Iyer[1], and Ben Langmead[1,*]

[1]Department of Computer Science, Johns Hopkins University
[*]*corresponding author;* langmea@cs.jhu.edu

November 24, 2020

Supplementary Notes

S1 RandFlow-LD variant set

We built a vg graph using the variant set selected by RandFlow-LD, including the major alleles and five superpop genomes, using `bcftools merge`. This alternative vg graph contains 6,471,383 variants across the whole GRCh38 (Table S3). The size of this set is comparable to the number of variants with $\geq$ 10% allele frequency and is less than one half of the number of variants with $\geq$ 1% allele frequency in the 1000 Genomes Project.

We further compared the allele frequency distribution of chromosome 21 variants in the RandFlow-LD set and the allele frequency $\geq$ 10% set (Figure S17). Because the RandFlow-LD method includes a major-allele reference, all variants with allele frequency greater than 50% are included in the set. There are 14,699 (15%) RandFlow-LD variants with allele frequencies in range $[0.1, 0.2)$, lower than 25,294 (28%) of the allele frequency $\geq$ 10% set. One fifth (19,279) of the RandFlow-LD variants have allele frequencies lower than 10%.

S2 RandFlow-LD variability

We assessed the variability of the RandFlow-LD method when using different random seeds. We generated 15 set of RandFlow-LD chr21 references, each containing five superpop genomes, using Python random seeds from 0 to 14. Then we repeated the experiments using simulated data, using identical setup as described in Sections 2.2 and 2.3. We compared the 15 RandFlow-LD results with vg using a graph built with all variants

with $\geq$ 1% allele frequency and showed that the variability of RandFlow-LD is very small compared to the variability in alignment methods in both alignment bias and allelic bias (Figure S5, S6 and S7).

Supplementary Figures

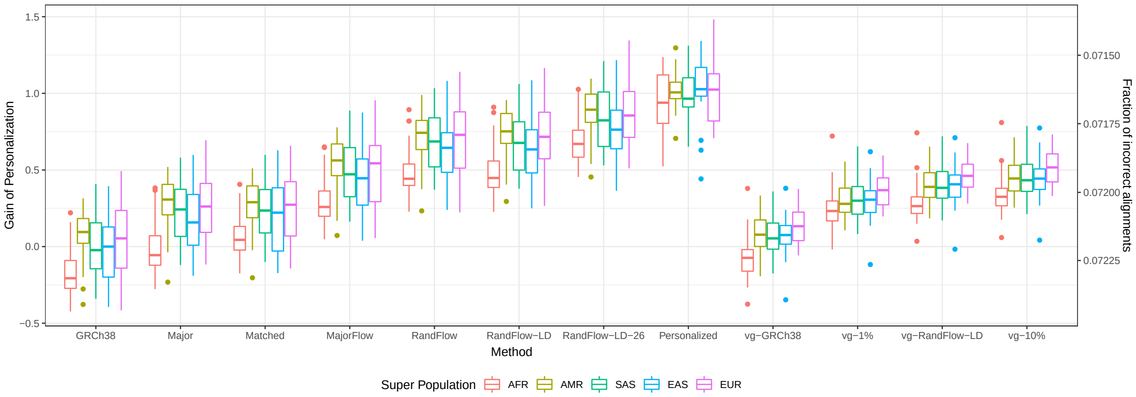

Figure S1: Number of incorrectly aligned reads when aligning 2M simulated reads. The experiment setup is identical to Figure 2 , so as results in columns *GRCh38*, *Major*, *Matched*, *MajorFlow*, *RandFlow*, *RandFlow-LD*, *RandFlow-LD-26* and *Personalized*. All columns with prefix "vg" use aligner vg. In the *vg-GRCh38* column reads are aligned to linear GRCh38; in the *vg-RandFlow-LD* column reads are aligned to a vg graph built using the RandFlow-LD variant set (Note S1); in the *vg-1%* and *vg-10%* columns reads are aligned to graphs built using variants with allele frequency $\geq$ 1% and 10% respectively. The columns are sorted by median sensitivity.

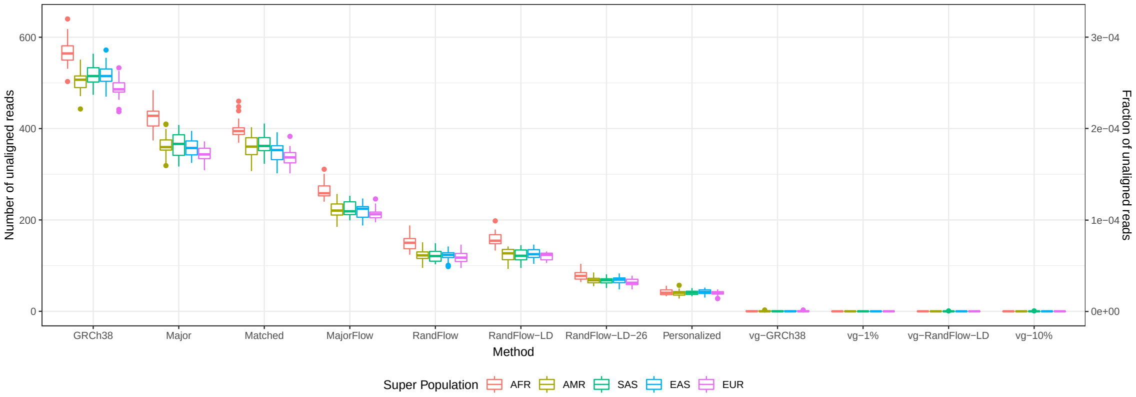

Figure S2: Number of unaligned reads when aligning 2M simulated reads. The experiment setup is identical to Figure S1.

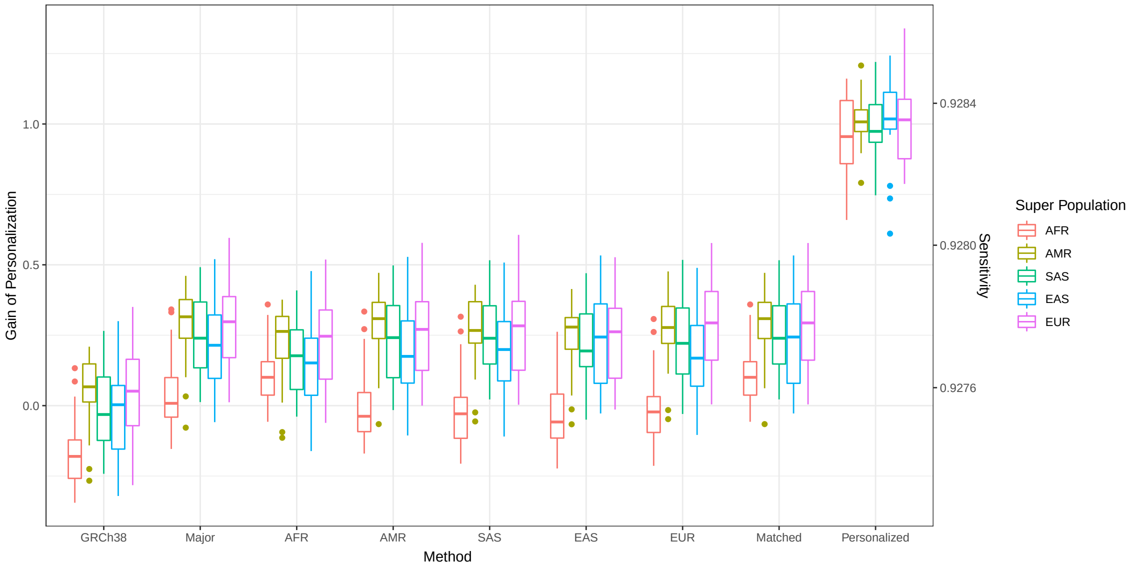

Figure S3: Population-stratified mapping sensitivity using typical methods that all reads are aligned to a single linear reference genome. 2M 100-bp single-end reads are simulated from chromosome 21 and aligned using Bowtie 2. 100 individuals (1M reads per haplotype, both SNVs and indels included) sampled from the 1000 Genomes Project are used for simulation. The *Major* column shows the results using the global major reference. Five superpop major references are labelled with the super population (*AFR*, *AMR*, *SAS*, *EAS* and *EUR*). In the *Matched* column reads are aligned to corresponding ethnicity-matching superpop major reference. The personalized reference genome (*Personalized*) is diploid and others are haploid.

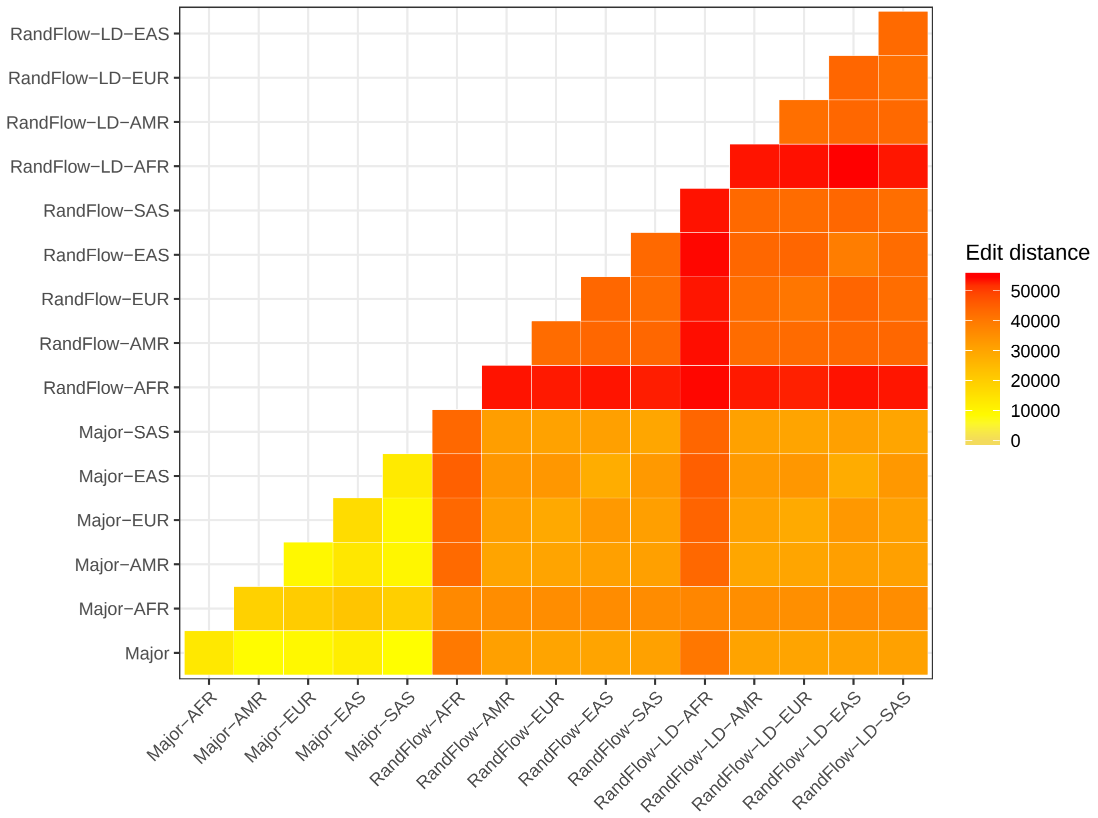

Figure S4: Edit distance, calculated by summing the number of edits for all variants using `bedtools` [1], between global major reference (*Major*), superpop major references (with *Major-* prefixes), stochastic super population genomes (independent-sampling: with *RandFlow-*, phase-preserving with 1000-bp blocks: with *RandFlow-LD-* prefixes).

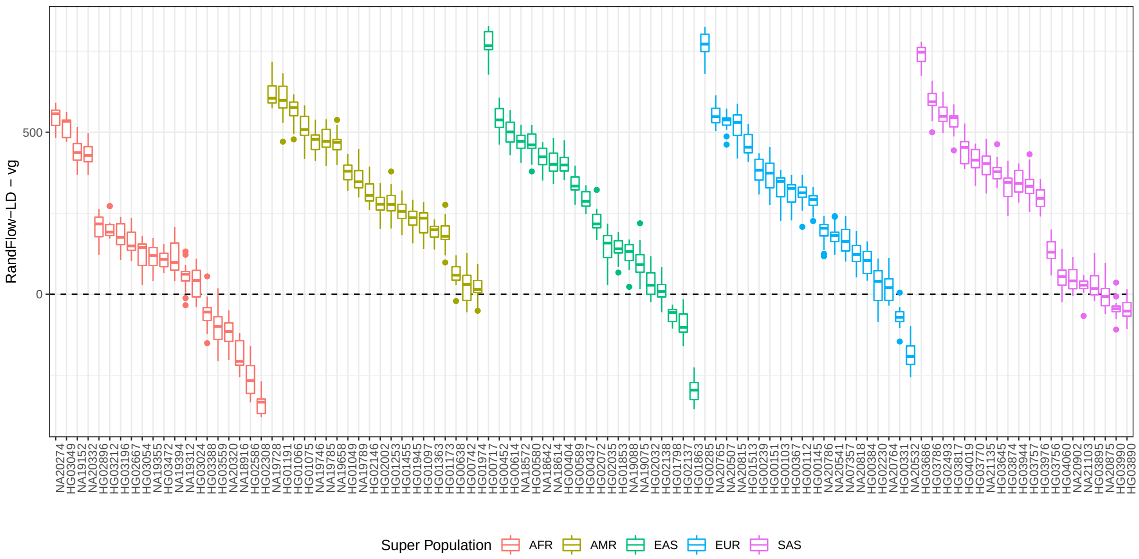

Figure S5: Difference in alignment sensitivity between using vg and 15 sets of RandFlow-LD references, each built with a distinct random seed. The dashed line represents equal sensitivity between two methods. Results within each super population are sorted by median difference in alignment sensitivity. The heights of the boxes convey variability due to the random seed used to select RandFlow genotypes, whereas the vertical spread of same-color boxes conveys variability due to genetic differences between donor individuals. Notably, variability due to random seed is substantially lower compared to variability due to donor-individual genetics.

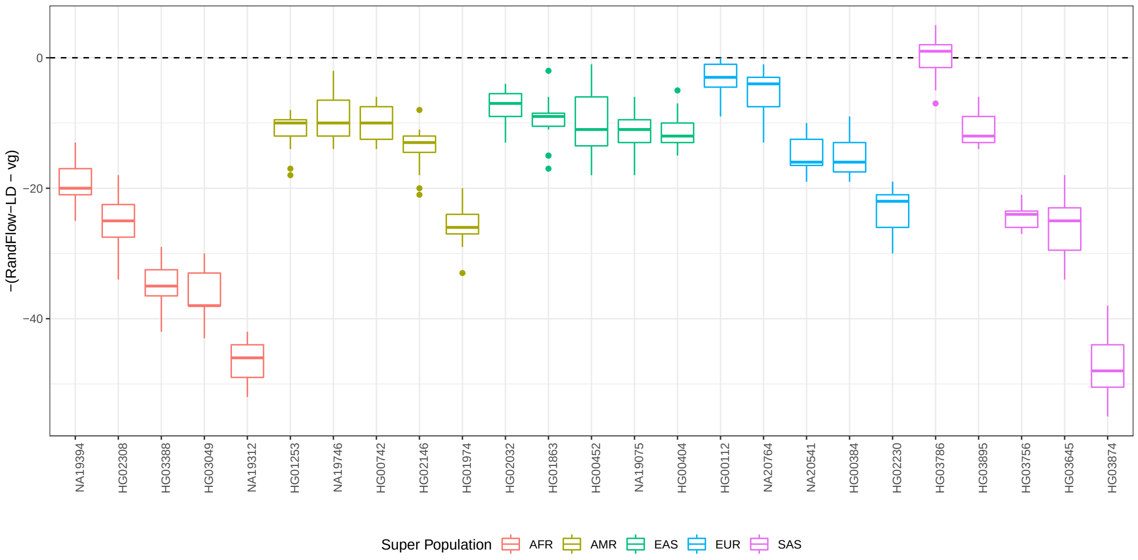

Figure S6: Difference in number of strongly biased sites between using vg and 15 set of RandFlow-LD references, each built with a distinct random seed. The dashed line represents zero difference between two methods. RandFlow-LD outperforms vg if the difference is positive (less number of biased sites). Results within each super population are sorted by median difference in number of strongly biased sites.

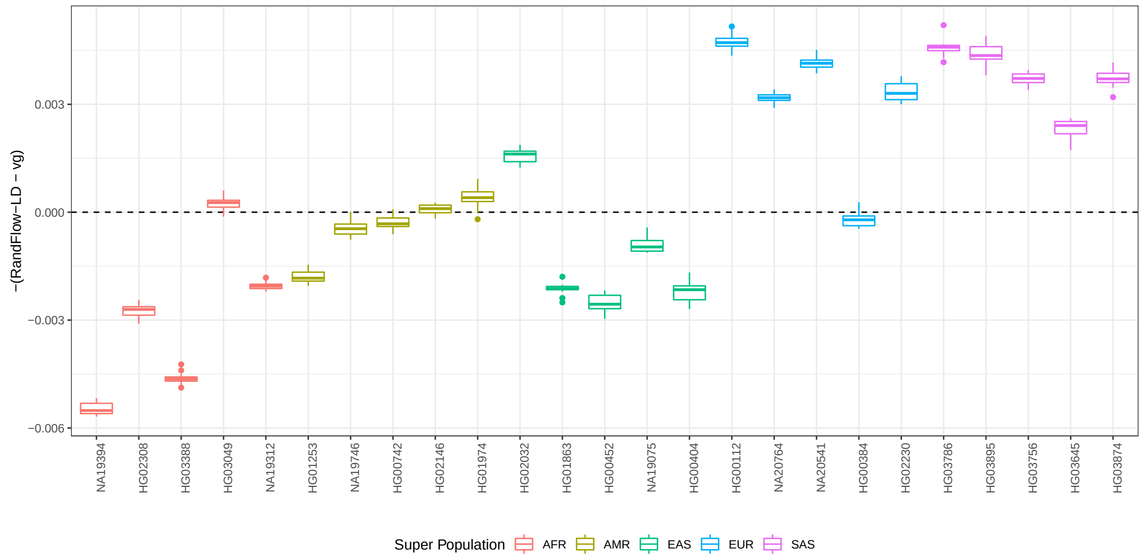

Figure S7: Difference in absolute REF-to-ALT ratio deviation (defined as $|1-\text{REF-to-ALT}|$) between using vg and 15 sets of RandFlow-LD references, each built with a distinct random seed. The dashed line represents equal REF-to-ALT ratios. RandFlow-LD outperforms vg if the difference is positive. Results within each super population are sorted by median difference in number of strongly biased sites to make it aligned with Figure S6.

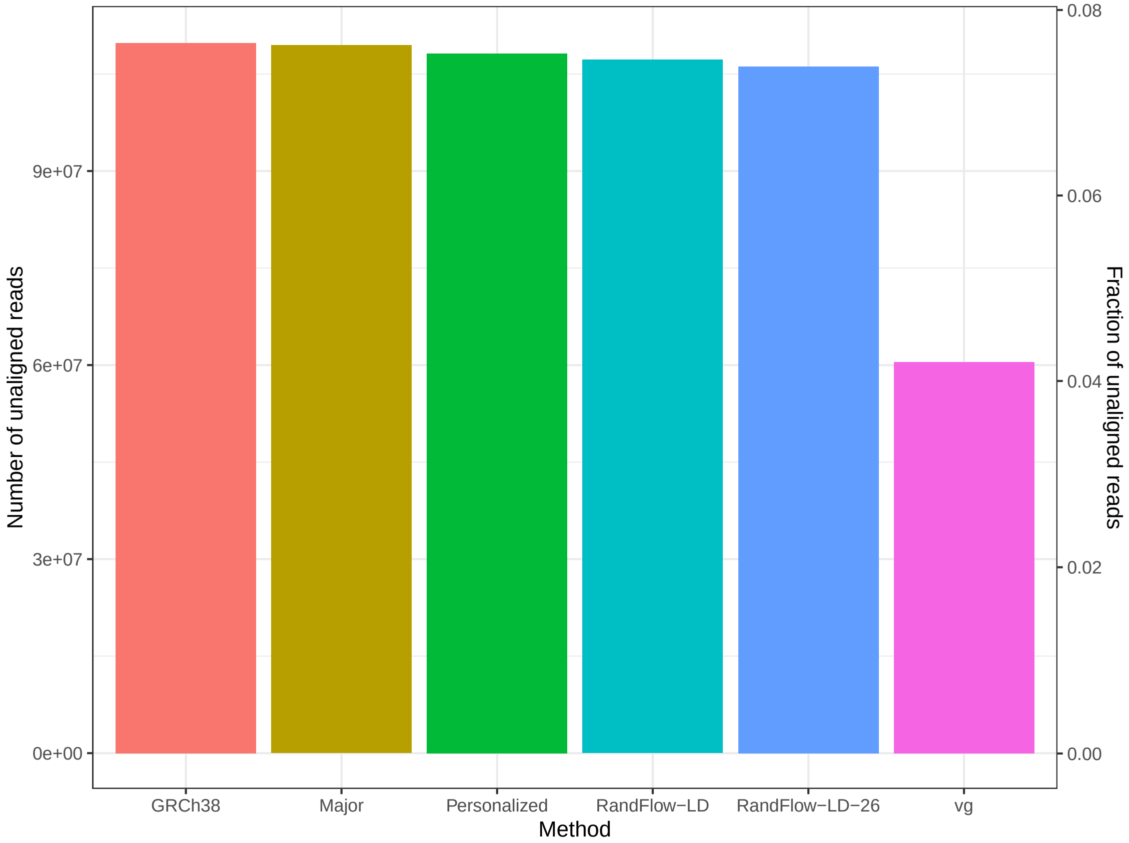

Figure S8: Number of unaligned reads when aligning real reads from SRR622457 to the whole-genome. The experiment setup is identical to Figure 3.

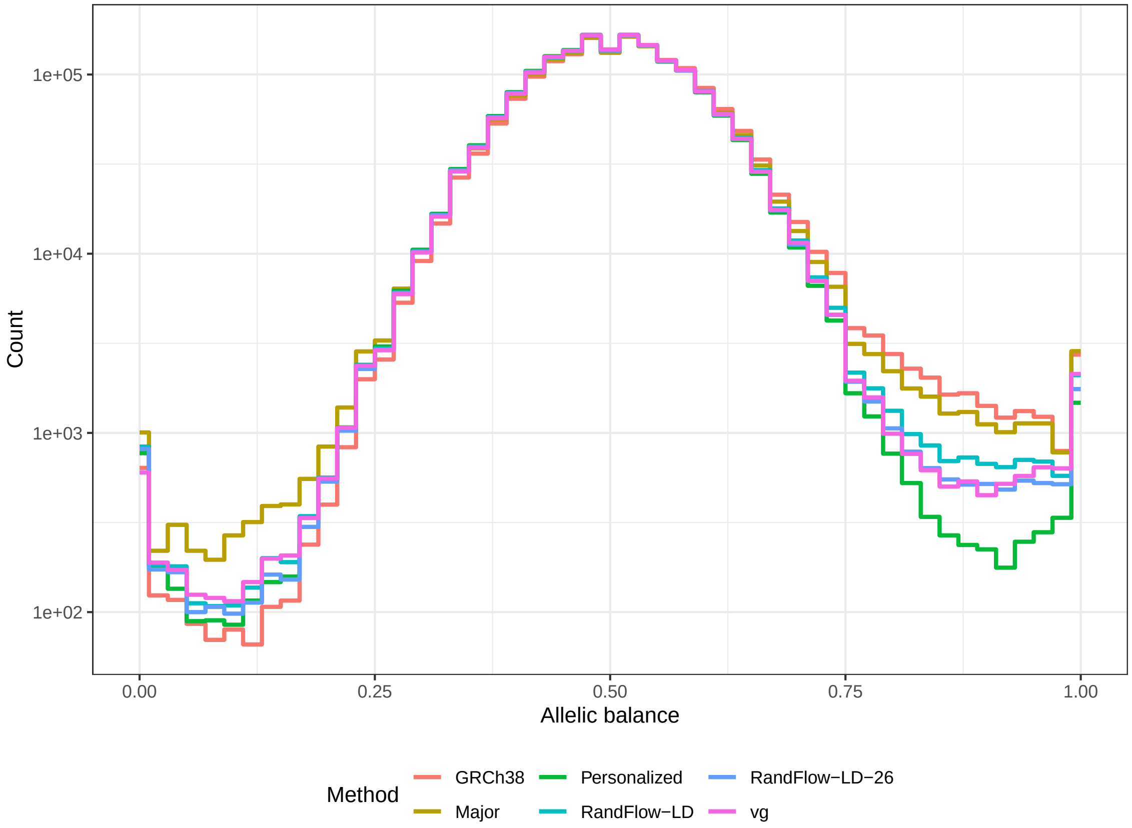

Figure S9: Histograms of allelic balance using a high-coverage real WGS dataset of individual NA12878 (SRR622457) in Genome-in-a-Bottle v3.3.2 high-confidence regions. Experiments are performed using GRCh38 (*GRCh38*), global major reference (*Major*), diploid personalized genome (*Personalized*), vg using alleles with frequency $\geq$ 10% (*vg*), reference flow using 1000-bp phased blocks with 5 super populations (*RandFlow-LD*) and reference flow using 1000-bp phased blocks with 26 populations (*RandFlow-LD-26*).

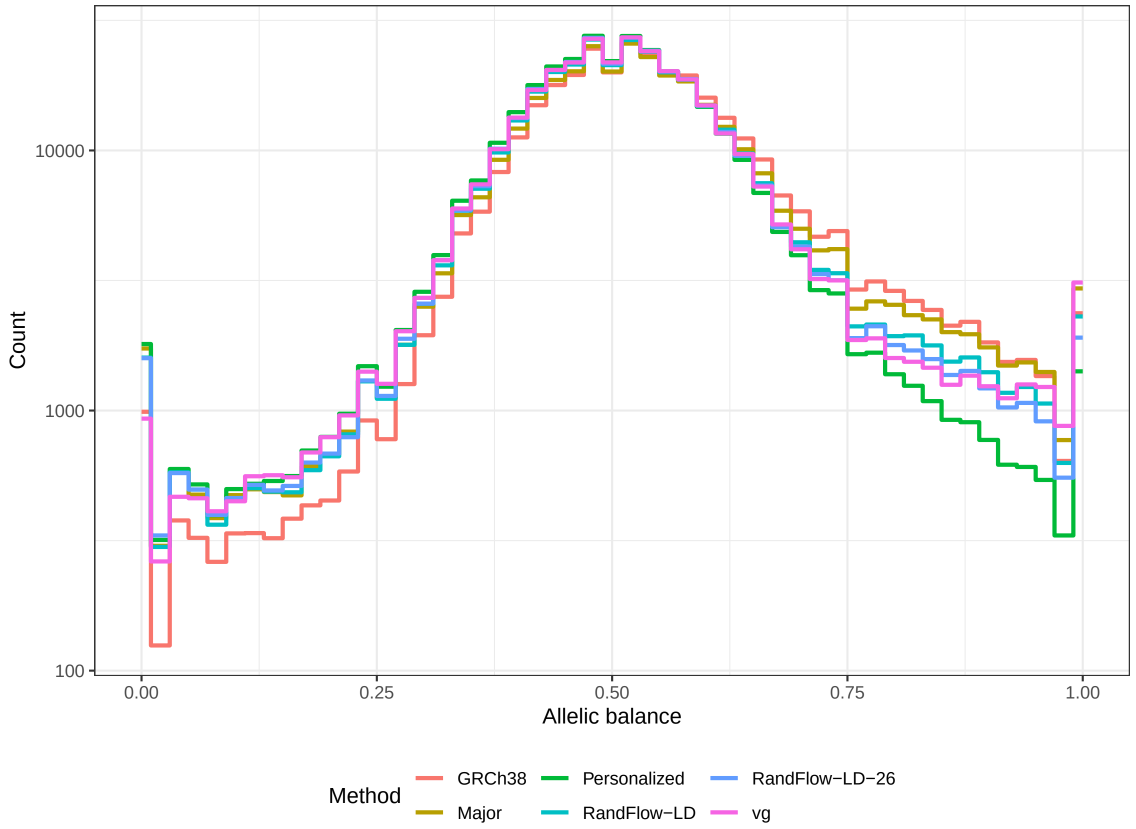

Figure S10: Histograms of allelic balance using a high-coverage real WGS dataset of individual NA12878 (SRR622457) in Genome-in-a-Bottle v3.3.2 low-confidence regions. Experiments are performed using GRCh38 (*GRCh38*), global major reference (*Major*), diploid personalized genome (*Personalized*), vg using alleles with frequency $\geq$ 10% (*vg*), reference flow using 1000-bp phased blocks with 5 super populations (*RandFlow-LD*) and reference flow using 1000-bp phased blocks with 26 populations (*RandFlow-LD-26*).

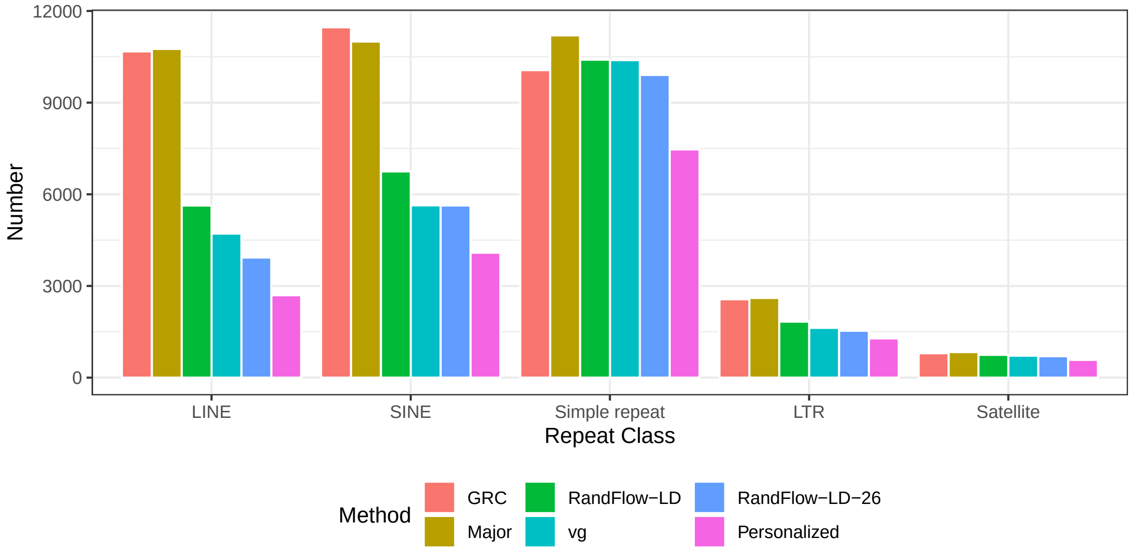

Figure S11: Number of strongly biased HET sites in repetitive elements across the NA12878 genome. Counts are stratified by RepeatMasker class[2]. The reads aligned are the first end of the paired-end reads in SRR622457. Variants with allele frequency $\geq 10\%$ in the 1000 Genomes Project are included in the vg graph.

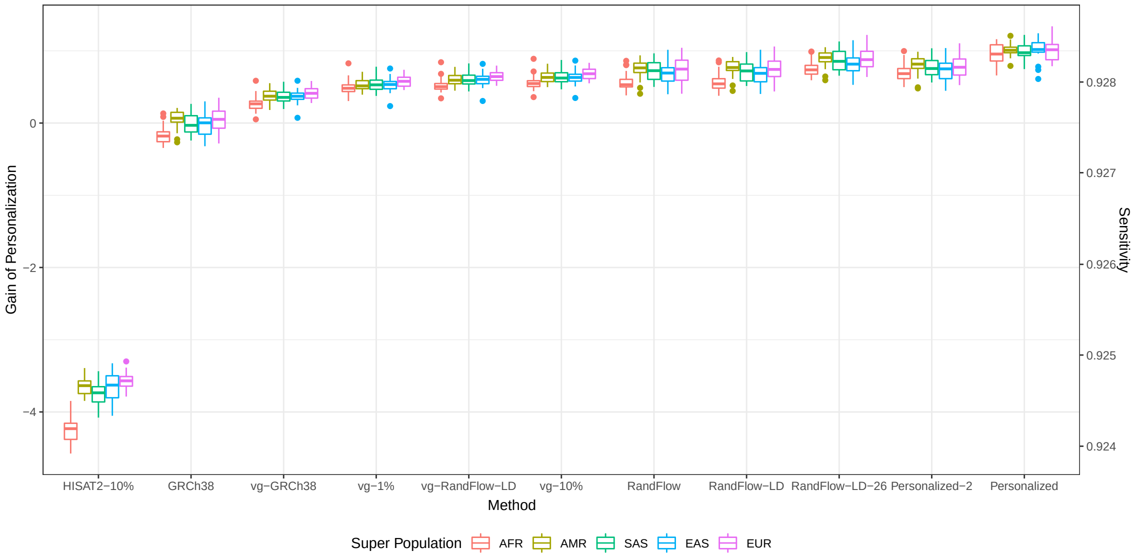

Figure S12: Population-stratified mapping sensitivity including various graph-based approaches. The experiment setup is identical to Figures 2a and S1, so as results in columns *GRCh38*, *RandFlow*, *RandFlow-LD*, *RandFlow-LD-26*, *vg-GRCh38*, *vg-RandFlow-LD*, *vg-1%*, *vg-10%* and *Personalized*. The *Personalized-2* column shows the results using each personalized haplotype once. The *HISAT-10%* column shows the results using HISAT2 with a graph built using all variants with $\geq$ 10% allele frequency in the 1000 Genomes Project. The columns are sorted by median sensitivity.

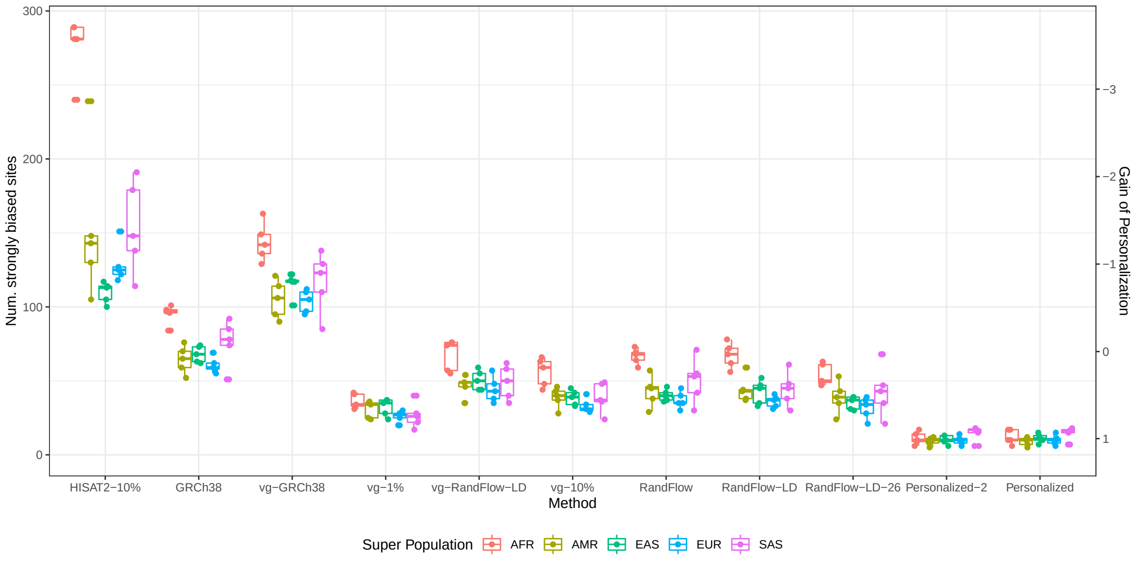

Figure S13: Population-stratified number of strongly biased HET sites including various graph-based approaches. The experiment setup is identical to Figure S12.

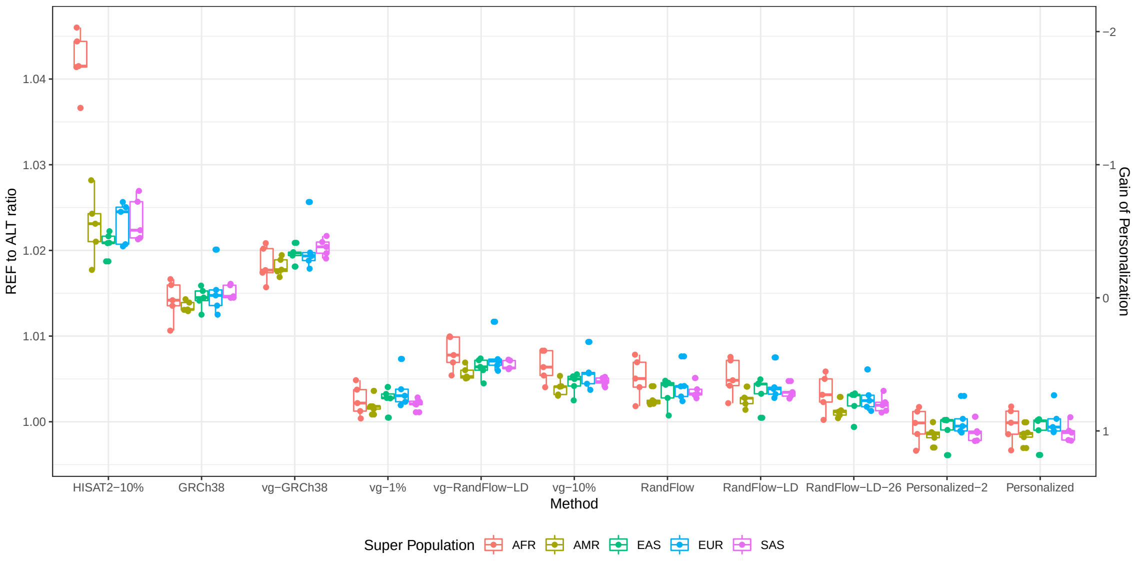

Figure S14: Population-stratified REF-to-ALT ratio including various graph-based approaches. The experiment setup is identical to Figure S12.

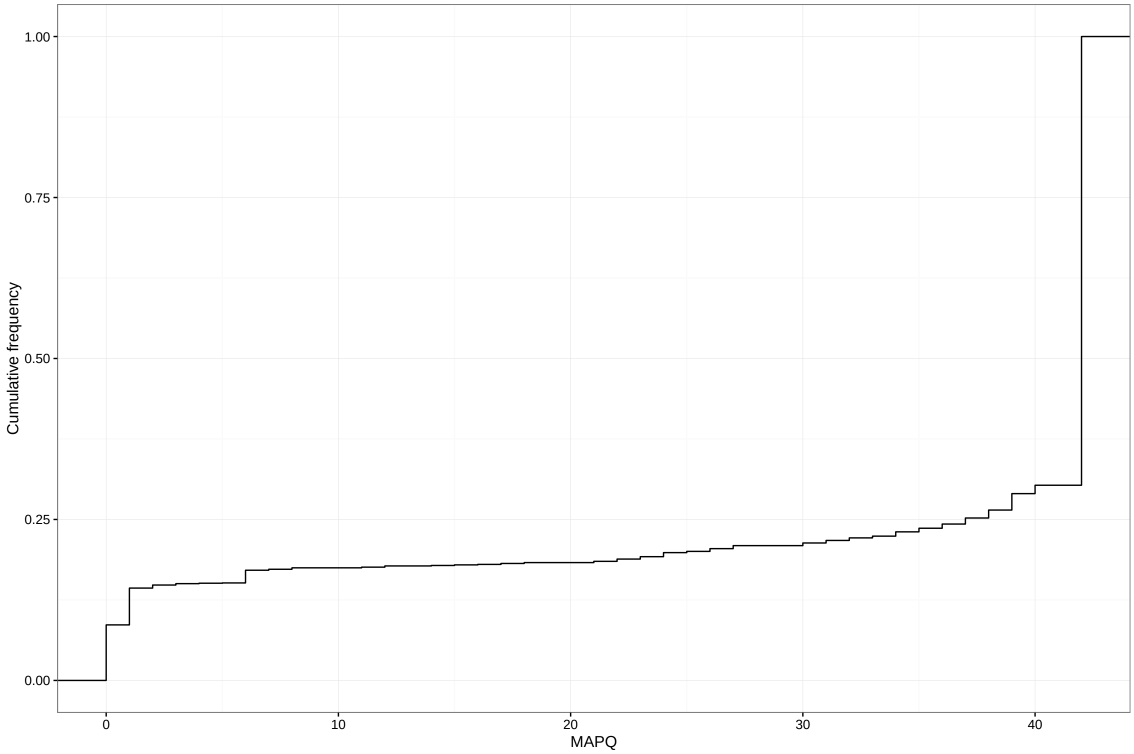

Figure S15: Cumulative frequency of mapping quality (MAPQ) when real whole genome sequencing reads from NA12878 (SRR622457) are aligned to the global major reference genome. More than 82% of reads are aligned with mapping quality $\geq$ 10.

We then simulated 2M 100-bp single-end reads using chromosome 21, with NA12878 genotypes, to evaluate alignment correctness. When the MAPQ threshold was set to 10, around 87% of reads were assigned into the committed group and more than 99.98% of these aligned correctly. The alignment sensitivity for the deferred group, where reads are unaligned or aligned with MAPQ lower than 10, was 44.93%.

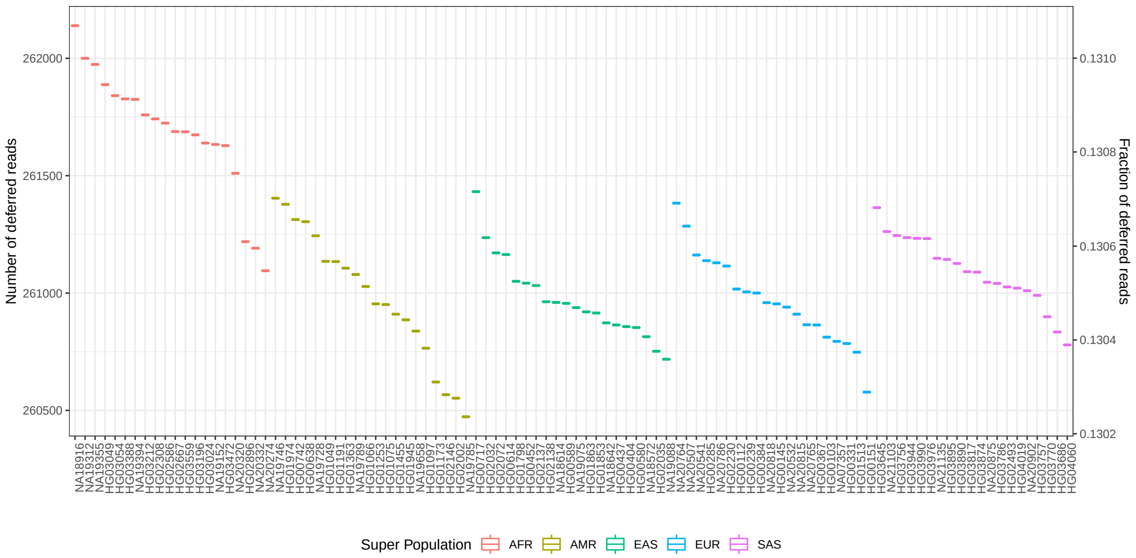

Figure S16: Number of deferred reads (unaligned in the first-pass or aligned with MAPQ ≥ 10) when aligning 2M simulated chr21 reads to the global major-allele reference. All reference flow methods discussed in this study — MajorFlow, RandFlow, RandFlow-LD and RandFlow-LD-26 — re-aligned the same set of deferred reads.

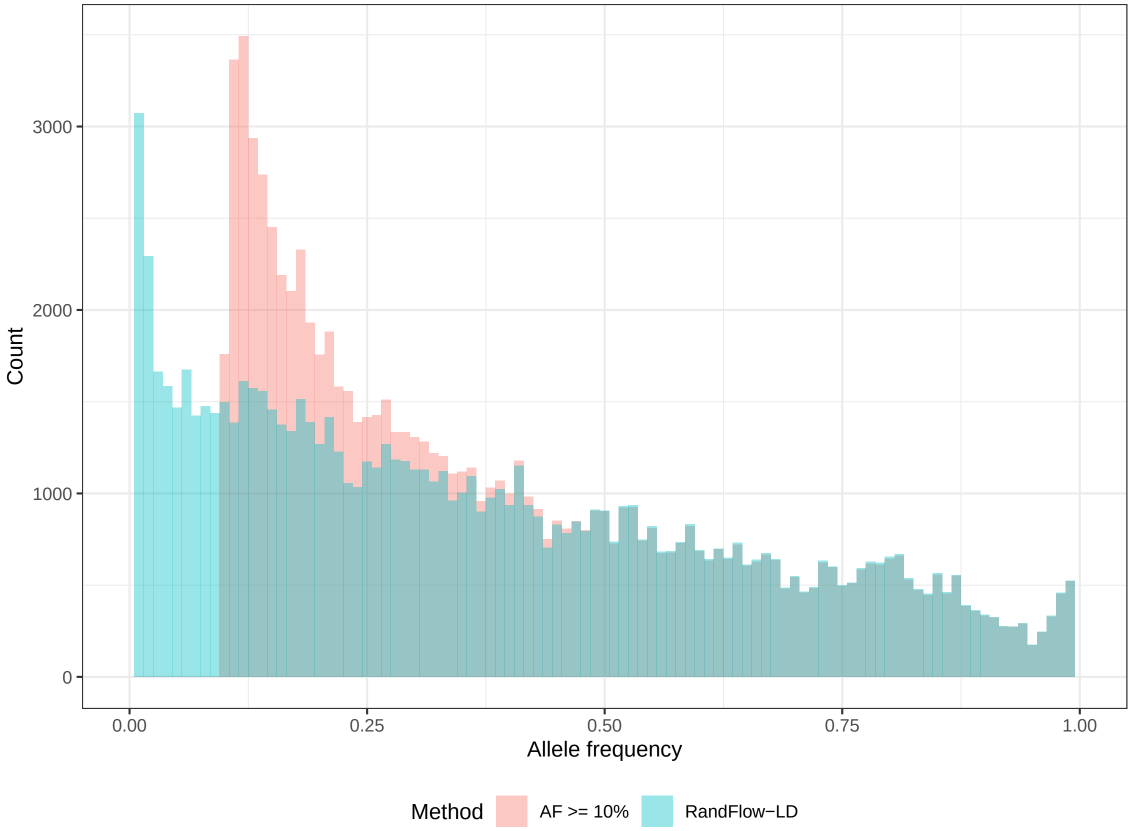

Figure S17: Allele frequency distributions of chromosome 21 RandFlow-LD variants and variants with allele frequency $\geq 10\%$. The RandFlow-LD variant set is the union of major alleles and five superpopulation RandFlow-LD variant sets.

Supplementary Tables

Table S1: A hundred individuals from the 1000 Genomes Project are randomly selected for the simulated experiment. Deeper datasets for allelic bias evaluation are simulated using individuals marked with boldface. Variant statistics for chromosome 21 are reported.

Sample	Superpopulation	Population	# variants	# SNP	# indel
**NA19312**	AFR	LWK	71930	63701	8229
**NA19394**	AFR	LWK	73097	64709	8388
**HG02308**	AFR	ACB	72263	63929	8334
**HG03049**	AFR	GWD	71053	62773	8280
**HG03388**	AFR	MSL	71986	63652	8334
HG03054	AFR	MSL	71155	62933	8222
NA19355	AFR	LWK	69661	61628	8033
HG02667	AFR	GWD	71042	62896	8146
HG03472	AFR	MSL	70196	62043	8153
HG02896	AFR	GWD	70096	62091	8005
NA18916	AFR	YRI	70866	62619	8247
HG03196	AFR	ESN	70081	62002	8079
HG03212	AFR	MSL	72923	64660	8263
NA20320	AFR	ASW	66891	59114	7777
HG02586	AFR	GWD	71779	63477	8302
NA20332	AFR	ASW	68034	60195	7839
NA20274	AFR	ASW	65038	57320	7718
NA19152	AFR	YRI	72300	64003	8297
HG03024	AFR	GWD	70341	62217	8124
HG03559	AFR	MSL	73777	65328	8449
**HG01974**	AMR	PEL	54457	47691	6766
**HG02146**	AMR	PEL	56570	49596	6974
**HG01253**	AMR	CLM	56730	49747	6983
**NA19746**	AMR	MXL	58632	51487	7145
**HG00742**	AMR	PUR	57494	50477	7017
HG00638	AMR	PUR	62005	54582	7423
HG02002	AMR	PEL	52990	46423	6567
NA19789	AMR	MXL	58615	51378	7237
HG01075	AMR	PUR	58322	51118	7204
HG01945	AMR	PEL	56900	50030	6870

Continued on next page

Table S1 – *Continued from previous page*

Sample	Superpopulation	Population	# variants	# SNP	# indel
HG01097	AMR	PUR	59376	52151	7225
NA19658	AMR	MXL	58734	51448	7286
HG01049	AMR	PUR	57367	50400	6967
HG01455	AMR	CLM	56625	49676	6949
NA19785	AMR	MXL	56906	49947	6959
HG01363	AMR	CLM	64792	57086	7706
HG01191	AMR	PUR	57277	50262	7015
NA19728	AMR	MXL	55754	48893	6861
HG01066	AMR	PUR	56643	49644	6999
HG01173	AMR	PUR	57606	50577	7029
**NA20541**	EUR	TSI	56993	50049	6944
**HG00384**	EUR	FIN	53008	46428	6580
**HG02230**	EUR	IBS	54943	48284	6659
**NA20764**	EUR	TSI	54921	48231	6690
**HG00112**	EUR	GBR	56087	49197	6890
NA07357	EUR	CEU	55650	48803	6847
HG00285	EUR	FIN	53313	46605	6708
NA20815	EUR	TSI	56315	49434	6881
HG00239	EUR	GBR	56735	49837	6898
HG00151	EUR	GBR	54968	48142	6826
HG00103	EUR	GBR	55580	48743	6837
NA20786	EUR	TSI	59886	52673	7213
NA20507	EUR	TSI	57635	50660	6975
NA20532	EUR	TSI	55229	48348	6881
HG00331	EUR	FIN	54977	48254	6723
NA20818	EUR	TSI	57044	50007	7037
HG00145	EUR	GBR	56072	49173	6899
HG00367	EUR	FIN	57514	50442	7072
HG01513	EUR	IBS	55368	48614	6754
NA20765	EUR	TSI	58726	51531	7195
**HG01863**	EAS	KHV	55137	48360	6777
**HG00404**	EAS	CHS	55263	48390	6873
**NA19075**	EAS	JPT	56286	49379	6907
**HG02032**	EAS	KHV	55801	48905	6896
**HG00452**	EAS	CHS	57862	50877	6985
HG01798	EAS	CDX	55114	48351	6763
NA18572	EAS	CHB	55384	48552	6832
HG02137	EAS	KHV	56923	50006	6917

Continued on next page

Table S1 – *Continued from previous page*

Sample	Superpopulation	Population	# variants	# SNP	# indel
HG00614	EAS	CHS	55597	48708	6889
HG00589	EAS	CHS	56125	49265	6860
HG00437	EAS	CHS	57259	50351	6908
HG02072	EAS	KHV	58807	51691	7116
NA19088	EAS	JPT	56111	49149	6962
HG01853	EAS	KHV	55178	48333	6845
NA18614	EAS	CHB	56190	49212	6978
HG02138	EAS	KHV	56748	49798	6950
HG00580	EAS	CHS	57456	50388	7068
NA18642	EAS	CHB	55687	48833	6854
HG00717	EAS	CHS	55592	48757	6835
HG02035	EAS	KHV	56565	49596	6969
**HG03756**	SAS	STU	58530	51352	7178
**HG03874**	SAS	ITU	58693	51625	7068
**HG03645**	SAS	STU	58696	51612	7084
**HG03895**	SAS	STU	50966	44682	6284
**HG03786**	SAS	ITU	58229	51133	7096
HG03817	SAS	BEB	56287	49307	6980
NA20902	SAS	GIH	57512	50511	7001
HG04060	SAS	ITU	56514	49641	6873
HG04019	SAS	ITU	57875	50819	7056
HG03770	SAS	ITU	59557	52338	7219
HG03990	SAS	STU	59801	52622	7179
HG03686	SAS	STU	57325	50370	6955
HG03757	SAS	STU	60150	52872	7278
NA21103	SAS	GIH	57945	50894	7051
HG03976	SAS	ITU	56991	49993	6998
NA21135	SAS	GIH	57912	50857	7055
HG02493	SAS	PJL	58844	51664	7180
HG03890	SAS	STU	58140	50998	7142
HG03944	SAS	STU	58349	51186	7163
NA20875	SAS	GIH	58039	51000	7039

Table S2: Allelic balance of the simulated NA12878 chr21 dataset. 20 million 100-bp single-end reads are aligned using different methods.

Method	# REF biases	# ALT biases	Total	REF to ALT ratio
GRCh38	70	1	70	1.0145
Major	52	16	70	1.0075
MajorFlow	48	13	59	1.0064
RandFlow-LD	32	11	44	1.0038
vg-1%	22	7	30	1.0026
Personalized	3	7	11	0.9992

Table S3: Numbers of variants across the whole-genome in augmented references. The RandFlow-LD set is the union of *Major* and five superpopulation RandFlow-LD variant sets.

Population	Number of variants
Personalized (diploid)	3,903,552
Major	1,998,961
RandFlow-LD	6,471,383
AFR (RandFlow-LD)	3,155,143
AMR (RandFlow-LD)	2,769,584
EAS (RandFlow-LD)	2,786,606
EUR (RandFlow-LD)	2,711,876
SAS (RandFlow-LD)	2,763,946
≥10% allele frequency	6,267,736
≥1% allele frequency	13,511,758

Table S4: Software versions used.

Software	Version
Bowtie 2[3]	2.3.4.3
vg[4]	1.19.0 "Tramutola"
Mason2[5]	2.0.0-beta1
bcftools[6]	1.9-206-g4694164
samtools[7]	1.9
Snakemake[8]	5.5.4
Python	3.7.3
GNU-time	1.9
R	3.6.2
GNU Parallel[9]	20160622

References

1. Quinlan, A. R. & Hall, I. M. BEDTools: a flexible suite of utilities for comparing genomic features. *Bioinformatics* **26,** 841–842 (2010).
2. Smit, A., Hubley, R. & Green, P. *RepeatMasker Open-4.0* `http://www.repeatmasker.org`. 2013-2015.
3. Langmead, B. & Salzberg, S. L. Fast gapped-read alignment with Bowtie 2. *Nature methods* **9,** 357 (2012).
4. Garrison, E., Sirén, J., Novak, A. M., Hickey, G., Eizenga, J. M., Dawson, E. T., Jones, W., Garg, S., Markello, C., Lin, M. F., *et al.* Variation graph toolkit improves read mapping by representing genetic variation in the reference. *Nature biotechnology* (2018).
5. Holtgrewe, M. Mason: a read simulator for second generation sequencing data (2010).
6. Li, H. A statistical framework for SNP calling, mutation discovery, association mapping and population genetical parameter estimation from sequencing data. *Bioinformatics* **27,** 2987–2993 (2011).
7. Li, H., Handsaker, B., Wysoker, A., Fennell, T., Ruan, J., Homer, N., Marth, G., Abecasis, G. & Durbin, R. The sequence alignment/map format and SAMtools. *Bioinformatics* **25,** 2078–2079 (2009).
8. Köster, J. & Rahmann, S. Snakemake – a scalable bioinformatics workflow engine. *Bioinformatics* **28,** 2520–2522 (2012).
9. Tange, O. *et al.* Gnu parallel-the command-line power tool. *The USENIX Magazine* **36,** 42–47 (2011).
