# Supplementary material for: Reference flow: reducing reference bias using multiple population genomes
Source: Genome Biol. 2021 Jan 4;22:8. doi: 10.1186/s13059-020-02229-3 (PMC7780692; doi:10.1186/s13059-020-02229-3)
Supplement: Supplementary file 2 — Additional file 2 Review history [file 13059_2020_2229_MOESM2_ESM.docx]

Review History

**First round of review**

**Reviewer 1**

**Were you able to assess all statistics in the manuscript, including the appropriateness of statistical tests used?** There are no statistics in the manuscript.

**Were you able to directly test the methods?** Yes.

**Comments to author:**

In this manuscript, Chen et al. describe a framework for ameliorating reference bias in read alignment by making use of a stage-wise "reference flow", where reads that align poorly to some reference (the standard reference or some universial major allele reference) are subsequently aligned against a small set of references constructed to represent different populations.  The idea is intuitive, the description is clear, and the manuscript is well-written).  It is, perhaps, a bit surprising how well this approach works compared to more complicated solutions like alignment to a genome graph using VG.  While I found the approach and results compelling overall, and likely to be of considerable use to those in the community for whom reference bias is a consideration in their analyses, I have a few questions and suggestions regarding the current manuscript.

Major
====

1) I found the idea of a "reference flow graph" to be both clever and elegant.  Therefore, it was a bit of a shame that it is not mentioned and introduced until the very end of the manuscript.  This is particularly important as the methods introduced by the authors all have "flow" in their names.  This reference to flow is lost without the introduction of the general reference flow graph idea.  I'd suggest the authors move that toward the beginning of the paper, and then explain that, in this manuscript, they explore only a "trivial" flow graph (i.e. one level of hierarchy).

2) The main contributions of the current paper seem to be (a) introducing the notion of reference flow (b) providing an algorithm to generate a good "cover" for various sub-populations (c) providing a tool / wrapper to allow implementing this reference flow idea with different underlying aligners in a modular way.  The comparison made with VG is comparing the linear alignment over a set of 6 (carefully chosen) references with VG alignment over a graph with > 192,000 distinct variants.  From this perspective, the comparison does not quite seem to be "apples-to-apples" when comparing the memory and time required by the different approaches.  I would recommend adding a variant of vg where the variant graph contains only those variants appearing in the ~6 references chosen in the RandFlow-LD set.  While this is obviously "handicapping" vg to some extent (it is considering a smaller set of variants), it allows comparing the performance of VG when filtering the variants in a much more intelligent way than simply discarding them based on frequency.  Also, it provides an alternative (and I believe, meaningful) comparison in memory and time.

3) Related to the above, it would be useful to also compare against HISAT2.  While HISAT2 is also a graph-based aligner in some sense, it claims to be much more memory and time efficient than VG, at least once the index has been constructed.  Also, since HISAT2 seems to already be quite widely-used (since it was around for quite a while before its initial publication), it seems like another relevant point of comparison.

4) The RandFlow and RandFlow-LD methods perform the best among those introduced by the authors.  However, as the name suggests, they are randomized.  Is it possible to provide some idea of the variability that might be expected between different random instantiations of the reference sets?  That is, if you ran the construction procedure a number of times seeding the pseudorandom number generator differently (or allowing it to be seeded from an external source), how much would the results change from run to run?  How typical are the results presented in the manuscript (presumably, they are close to the mean, but it would be good to know what type of variation can occur)?  I am not sure how feasible it is to build a "confidence interval", given the processing involved in the pre-processing phase here, but at least a few random instances could be provided to give a rough idea of the variation that could be expected.

5) I'm a bit uncomfortable with the wording "Our results also reinforce that a well configured linear aligner can exhibit similar accuracy to a graph aligner."  I would not consider the reference flow framework, and the RandFlow(-LD) algorithms to be "configurations" of a linear aligner.  They are aligning to a series of (intelligently constructed) linear genomes, and  are using a workflow that is far beyond the scope of any off-the-shelf linear aligner.  While I agree that the results demonstrate that using a linear aligner in conjunction with a "meta algorithm" like RandFlow(-LD) can exhibit similar accuracy to a graph aligner, this is not as simple as configuring the linear aligner and, in fact, a lot of extra work is being done.


Potentially interesting suggestion (doesn't seem essential to address if the authors don't desire):

* To what extent might it be possible to derandomize the RandFlow(-LD) construction?  Currently, a sampling based approach is being taken to yield a representative genome per-population, but could this sampling be made deterministic?  Alternatively, could one use another sampling approach to "cover" the reference space better.  This would provide a deterministic process for building the reference set and, perhaps, there is even a covering criterion one could optimize that is correlated with the performance of the approach in terms of reducing reference bias.


Minor
====

6) The very last sentence of the manuscript (lines 16-18 on page 14) seemed to have some missing words / typos.

7) Please provide a license for the ref-flow software in the repository.

**Reviewer 2**

**Were you able to assess all statistics in the manuscript, including the appropriateness of statistical tests used?** Yes, and I have assessed the statistics in my report.

**Were you able to directly test the methods?** No.

**Comments to author:**

The authors present a short-read alignment strategy that relies on
linear, and not graph-based reference genomes. In a first step, reads
are aligned against a standard "major allele" reference genome, which
differs from the ordinary reference genome by placing major alleles at
all polymorphic sites. In a second step, reads that did not align at
sufficient quality, or didn't align at all, are aligned against five
more linear reference genomes. Each of these represents one of the five
super populations labeled by the 1KG project. Again, at each
polymorphic site the allele that is predominant (major) in the
respective population is inserted. The corresponding method
is referred to as 'MajorFlow'.

The authors further study an alternative strategy, where alleles for
polymorphic sites are randomly drawn, according to the probability at
which they occur in the (super) population. The method is called
'RandFlow'. Furthermore, the authors do not sample alleles
individually, but sample entire haplotype blocks (of length 1000 bp),
and insert the sampled block into the super population reference
genome; the corresponding method is called 'RandFlow-LD'.

The major competitor of the strategies presented is 'vg', which
reflects the state of the art when seeking to compute graph-based
short-read alignments.

Rather unsurprisingly, because I have seen similar such results
before, which were concerned with comparing more sophisticated linear
reference alignment strategies with graph-based alignment strategies,
the new methods 'MajorFlow', 'RandFlow' and 'RandFlow-LD' outperform
'vg' in terms of compute resource requirements (quite substantially),
while 'vg' keeps (small, but still marked) advantages in terms of the
quality of the alignments computed. While MajorFlow does not lead to
increased sensitivity when aligning reads, and suffers in particular
when dealing with the (the most diverse) African super population,
RandFlow and RandFlow-LD are rivaling 'vg' in this respect. Still,
RandFlow and RandFlow-LD suffer from (certainly significantly) more
allele bias relative to the reads they are able to align than 'vg'.

I found the study interesting to a certain degree: it puts a
particular focus on how one can integrate the 1KG super populations
when trying to profit from resource friendly linear reference
alignment technology.

However, it also remained unclear to me to what degree super
populations really can enhance linear alignment strategies, because
the results do not necessarily look uncommon to me in comparison to
alternative linear reference based alignment strategies. For example,
it seems to become (ever more) obvious that when dealing with reads
from genomes sufficiently close to the common reference genome (like
reads from non-African super populations), linear reference based
strategies may already be a sufficiently satisfying option, which is
not necessarily surprising.

Overall, the study left me behind with the following substantial
questions:

* Why should randomly picking alleles, instead of using major alleles
 lead to advantages? Couldn't the randomly composed genomes just
 reflect statistical outliers? The authors should repeat the
 procedure of randomly picking alleles several times, also because
 the amount of reads used is rather small, and refers to only one of
 the chromosomes.

* How would 'vg' behave, if the exact same alleles that were picked
 for the generation of the linear references used here, were inserted
 into the variation graphs? The way experiments are designed does not
 reflect a true head-to-head comparison. Of course, however, the way
 'vg' is used is perfectly fine and reflects a standard strategy.

 To my understanding, choosing 6 genomes and aligning against them
 linearly, by using linear alignment tools, reflects to consider a
 variation type graph that hosts the variants incorporated into the 6
 genomes and specifying the paths that reflect the 6 genomes along
 with the graph structure when aligning against the graph.

* 'RandFlow(-LD)' can be interpreted as using the major allele
 reference, together with five more genomes, each of which sampled
 from alleles characterizing one of the 5 super populations.

 What I would find a truly interesting idea is to not only pick 5
 randomly (and by population constraints consistently) sampled
 genomes, but increase the amount of randomly sampled such
 genomes. One would then further explore the limits of such a
 strategy: at which number of (by population consistently picked)
 random genomes would advantages in terms of compute resource
 expenses disappear (in comparison to 'vg'), and, at the same time,
 would one possibly truly rival the performance of 'vg' in terms of
 alignment quality/biases?

 In other words, this could shed light what the trade-off between
 compute resource requirements on the one hand and alignment quality
 on the other hand looks like when trying to maximally increase
 alignment quality, while adding randomly sampled genomes, one by
 one. In other words: how does alignment quality behave plotted
 against increasing costs in terms of resources.

 Possibly, the linear alignment strategy is better behaved with
 respect to the balance of resource costs and alignment performance.
 
* While results are concerned with sensitivity, they are never
 concerned with false positives, i.e. the amount of incorrectly
 placed alignments.  It would be interesting to understand how many
 not correctly placed alignments were indeed incorrectly placed (and
 hence are false positives) in comparison to reads that remained
 entirely unaligned. One could consider incorrect placement worse
 than refusing to align reads at all, because suggesting incorrect
 placements could lead to flawed conclusions in downstream analyses.


There are some further major concerns:

* The entire paper reads like a case study. There are not necessarily
 any truly novel methodical achievements other than an alignment
 protocol.

* One of the major advantages of 'vg' is to reflect a coordinate system
 that applies for several genomes together. How much time does one
 loose in the methods presented here for unifying alignments?

* RandFlow and RandFlow-LD require to generate genomes by random
 principles. The generation of the respective genomes also costs
 time. To what degree does the generation of these genomes play a
 role when considering runtime? Or is this just completely
 negligible? Possibly also fixing the sampled genomes once and
 forever; but then how can one make sure that the random genomes do
 not reflect outliers that lead to bad alignment quality on at least
 some of the chromosomes?

MINOR:

* Citation 10 (which I thoroughly read earlier, and not to my
 surprise, still is not published) -- to the best of my understanding
 -- does not justify by itself to pursue reference based approaches,
 instead of graph based approaches. Many of the analyses seem to be
 flawed because of the small amounts of data used. In my opinion, the
 baseline method seems to be rather contrived and to my judgment is
 not necessarily a *baseline* method. The advantages on reference
 reads when comparing with graph based approaches cannot be
 considered an advantage when trying to overcome issues when trying
 to align reads that harbor non-reference variation. So I would
 possibly use additional citations in the hunt for evidence that
 linear reference based alignment strategies could have advantages.
 (see https://genomebiology.biomedcentral.com/articles/10.1186/s13059-020-01963-y
 for example)

Intro:

- p2, l29: why 'rough' upper bound? Isn't this the ideal scenario for
 a linear reference aligner, so just the 'ultimate' upper bound? (am
 I overlooking something here?)

Discussion:

- p14, l16: "In principle are not difficult to include..." (broken
 sentence)

**Reviewer 3**

**Were you able to assess all statistics in the manuscript, including the appropriateness of statistical tests used?** There are no statistics in the manuscript.

**Were you able to directly test the methods?** No.

**Comments to author:**

My overall impression is that this is a good study, the method is a reasonable tradeoff in time and memory comparedto state of the art, vg. However the results of the experiment show minor improvements in sensitivity, and the ref to alt ratio goes from 1.5% bias down to 0.5% bias.

The results as presented are a bit underwhelming almost raising the question of "why bother". What I think needs to be communicated properly is that the average is hiding more serious artefacts that can cause trouble in downstream analysis and interpretation. To do this I would recommend working with a specific example in the introduction, e.g. from RNA-seq or CHIP-seq (e.g. like this paper https://genomebiology.biomedcentral.com/articles/10.1186/s13059-020-02038-8), where a misalignment bias can flip a result. If the focus is supposed to be on sequencing of DNA the next obvious step is variant calling, how are these sites affected, e.g. by running GATK on them?

I would also recommend running this on real data from GIAB which is our best approximation for a gold standard


Comments

page 2, line 13
not clear whether misalignment or unalignment, also mapping vs alignment

page 3, line 12
Not clear which reads, "all reads" are, only later becomes clear they are simulations

page 3, figure 1,a,b
Matched is missing from b,c, no explanation in legend
Cross between vg and Randflow-LD, hard to compare, keep the order
Sensitivity ranges from 0.9275 to 0.9285, this is 1000 reads out of 1M.

page 6, lines 33-40
Use of real data is good, but can be improved by using genome in a bottle on the same sample, trios for NA12878 and a "truth" set of variants are available, while not as good as a simulation you get real datasets.
Also, GIAB, has a high confidence region defined, covers 70% of the genome, in which short reads are "easy", it would be interesting to see the performance of the tools on this regions vs outside.

page 6, line 46
basied, typo

page 8, line 44
8% of the CPU
It is not clear at this point (or in the paper) that vg was only run on the same reads as the majorflow, this should be laid out clearly at the start.

What is the effect of running vg on the entire read set, how close is it to the personalized alignments?


page 10, line 24
for 0.2-0.8, Given an average coverage of 30x and assuming a Poisson coverage, what is the probability of observing this by chance given a perfectly unbiased sample.


I would recommend adding a table, supplementary or in the paper, with the following statistics
What fraction of the simulated reads were unaligned and which were poorly aligned.

For each method
How many of the poorly aligned reads were mapped to different regions with your method.
How many of the unaligned were mapped to a unique region in the genome

**Authors Response**

**Point-by-point responses to the reviewers’ comments:**

Reviewer #1:

In this manuscript, Chen et al. describe a framework for ameliorating reference bias in read alignment by making use of a stage-wise "reference flow", where reads that align poorly to some reference (the standard reference or some universial major allele reference) are subsequently aligned against a small set of references constructed to represent different populations. The idea is intuitive, the description is clear, and the manuscript is well-written). It is, perhaps, a bit surprising how well this approach works compared to more complicated solutions like alignment to a genome graph using VG. While I found the approach and results compelling overall, and likely to be of considerable use to those in the community for whom reference bias is a consideration in their analyses, I have a few questions and suggestions regarding the current manuscript.

Major

====

1) I found the idea of a "reference flow graph" to be both clever and elegant. Therefore, it was a bit of a shame that it is not mentioned and introduced until the very end of the manuscript. This is particularly important as the methods introduced by the authors all have "flow" in their names. This reference to flow is lost without the introduction of the general reference flow graph idea. I'd suggest the authors move that toward the beginning of the paper, and then explain that, in this manuscript, they explore only a "trivial" flow graph (i.e. one level of hierarchy).

==response begin==

We agree that the idea should be described in the introduction, before the results and before the more detailed treatment in the Discussion. We addressed this in two ways. First, we added the following sentences to the introduction:

--

We call the method "reference flow" because it involves the selective use of different reference genomes according to how well reads aligned to the previous ones tried. In this work, we propose specific reference-flow strategies where the method proceeds in two passes… (followed by original text)

–

Second, we moved the old Figure 3 to be Figure 1, now referenced in the first added sentence. We adapted the figure to more clearly describe the general reference-flow strategy: the decision-making process for how reads get forwarded from one genome to the next, and how to merge alignments to produce the final BAM file. The caption was rewritten to further explain how the "two-pass" methods we implement could be iterated further. Together with language that was already in the manuscript (e.g. the "we can also consider a wider class of possible architectures" paragraph in the discussion), we now provide a clearer way of thinking about the overall framework and how our RandFlow approaches fit in. ==response end==

2) The main contributions of the current paper seem to be (a) introducing the notion of reference flow (b) providing an algorithm to generate a good "cover" for various subpopulations (c) providing a tool / wrapper to allow implementing this reference flow idea with different underlying aligners in a modular way. The comparison made with VG is comparing the linear alignment over a set of 6 (carefully chosen) references with VG alignment over a graph with > 192,000 distinct variants. From this perspective, the comparison does not quite seem to be "apples-to-apples" when comparing the memory and time required by the different approaches. I would recommend adding a variant of vg where the variant graph contains only those variants appearing in the ~6 references chosen in the RandFlow-LD set. While this is obviously "handicapping" vg to some extent (it is considering a smaller set of variants), it allows comparing the performance of VG when filtering the variants in a much more intelligent way than simply discarding them based on frequency. Also, it provides an alternative (and I believe, meaningful) comparison in memory and time.

==response begin==

We agree that the work is strengthened by adding this more "apples to apples" comparison. As suggested, we compiled the set of variants used by the RandFlow-LD references, containing the union of the major alleles (used for first-pass alignments) and the variants used in five superpop references (used for second-pass). We built a vg graph with this variant set and evaluated its performance on chr21, setting up the experiments in the same way as the original Section 2.2. We showed that using RandFlow-LD achieved both lower bias and higher alignment sensitivity, compared to vg using the RandFlow-LD variants. We also evaluated different vg settings including using a linear genome, an Allele Frequency (AF) >=1% graph, the RandFlow-LD graph, and an AF>=10% graph.

All of these new results are discussed in the new results Section 2.6. We compared the allele frequency distribution between the RandFlow-LD variant set with the AF>=10% set in Note S2 and Table S5. Results using the vg-RandFlow-LD graph and other graph-based methods are shown in Figures S6-8. ==response end==

3) Related to the above, it would be useful to also compare against HISAT2. While HISAT2 is also a graph-based aligner in some sense, it claims to be much more memory and time efficient than VG, at least once the index has been constructed. Also, since HISAT2 seems to already be quite widely-used (since it was around for quite a while before its initial publication), it seems like another relevant point of comparison.

==response begin==

We agree that the work is strengthened by comparing to the popular HISAT2 aligner. We performed experiments using HISAT2 and a graph built from variants with >= 10% allele frequency in the 1000 Genomes Project. We added a subsection in Results which showed that HISAT2 had better computational efficiency even than Bowtie2, but lower accuracy, consistent with other studies. The results are similar to those reported by Grytten et al.

We now discuss these results in the first paragraph of the new Section 2.6 ("Comparison of variant-aware alignment approaches"). Results based on HISAT2 and other graph-based methods are shown in Figures S6-8. ==response end==

4) The RandFlow and RandFlow-LD methods perform the best among those introduced by the authors. However, as the name suggests, they are randomized. Is it possible to provide some idea of the variability that might be expected between different random instantiations of the reference sets? That is, if you ran the construction procedure a number of times seeding the pseudorandom number generator differently (or allowing it to be seeded from an external source), how much would the results change from run to run? How typical are the results presented in the manuscript (presumably, they are close to the mean, but it would be good to know what type of variation can occur)? I am not sure how feasible it is to build a "confidence interval", given the processing involved in the pre-processing phase here, but at least a few random instances could be provided to give a rough idea of the variation that could be expected.

==response begin==

We agree that it would put the method on more solid ground if we could measure the variability induced by the random choices of allele. In particular, we thought about how to compare this variability to other sources of variability, such as between random donor individuals and between tools.

To achieve this, we built 15 sets of RandFlow-LD chr21 references using different random seeds, yielding different allelic choices. We re-ran the chr21 simulation experiment for each. As we did in Figure 2, we ran alignments using these 15 RandFlow-LD references on 100 samples for measuring alignment sensitivity and on 25 deeper samples for measuring allelic bias metrics. We now describe these new results in Note S2, Figure S10-12. We further compared the results with vg’s. To excerpt the caption of Figure S10: "variability due to random seed is substantially lower compared to variability due to donor-individual genetics." ==response end==

5) I'm a bit uncomfortable with the wording "Our results also reinforce that a well configured linear aligner can exhibit similar accuracy to a graph aligner." I would not consider the reference flow framework, and the RandFlow(-LD) algorithms to be "configurations" of a linear aligner. They are aligning to a series of (intelligently constructed) linear genomes, and are using a workflow that is far beyond the scope of any off-the-shelf linear aligner. While I agree that the results demonstrate that using a linear aligner in conjunction with a "meta algorithm" like RandFlow(-LD) can exhibit similar accuracy to a graph aligner, this is not as simple as configuring the linear aligner and, in fact, a lot of extra work is being done.

==response begin==

We agree that reference flow and other pan-genome methods could be seen as going well beyond a "configuration" of a linear aligner. We changed the sentence to “Our results also reinforce that a linear aligner can be extended to incorporate variants and exhibit similar accuracy to a graph aligner.” So the notion of "extension" replaces the notion of "configuration." ==response end==

Potentially interesting suggestion (doesn't seem essential to address if the authors don't desire):

* To what extent might it be possible to derandomize the RandFlow(-LD) construction? Currently, a sampling based approach is being taken to yield a representative genome per-population, but could this sampling be made deterministic? Alternatively, could one use another sampling approach to "cover" the reference space better. This would provide a deterministic process for building the reference set and, perhaps, there is even a covering criterion one could optimize that is correlated with the performance of the approach in terms of reducing reference bias.

==response begin== While we showed that the variability due to the randomness of RandFlow-LD is small (Note S2, Figures S10-12), we agree that it is of interest to derandomize the references, or to provide a more efficient method to cover the space better. We also agree that such an alternative would likely be a method that optimizes an objective, where the objective is to "cover" the space well. We added a new paragraph to our Discussion section outlining a few ideas and frameworks that could enable this. The paragraph discusses using unsupervised, sequence-driven clustering methods, the "founder sequence" framework, submodular optimization approaches, as well as the more radical idea of simply indexing all haplotypes without choosing representatives. ==response end==

Minor

====

6) The very last sentence of the manuscript (lines 16-18 on page 14) seemed to have some missing words / typos.

==response begin==

Thank you for pointing this out. We corrected it. ==response end==

7) Please provide a license for the ref-flow software in the repository.

==response begin==

The reference flow software is under the MIT license, and the license is now included in the repo: https://github.com/langmead-lab/reference_flow/blob/master/LICENSE ==response end==

Reviewer #2:

The authors present a short-read alignment strategy that relies on linear, and not graph-based reference genomes. In a first step, reads are aligned against a standard "major allele" reference genome, which differs from the ordinary reference genome by placing major alleles at all polymorphic sites. In a second step, reads that did not align at sufficient quality, or didn't align at all, are aligned against five more linear reference genomes. Each of these represents one of the five super populations labeled by the 1KG project. Again, at each polymorphic site the allele that is predominant (major) in the respective population is inserted. The corresponding method is referred to as 'MajorFlow'.

The authors further study an alternative strategy, where alleles for polymorphic sites are randomly drawn, according to the probability at which they occur in the (super) population. The method is called 'RandFlow'. Furthermore, the authors do not sample alleles individually, but sample entire haplotype blocks (of length 1000 bp), and insert the sampled block into the super population reference genome; the corresponding method is called 'RandFlow-LD'.

The major competitor of the strategies presented is 'vg', which reflects the state of the art when seeking to compute graph-based short-read alignments.

Rather unsurprisingly, because I have seen similar such results before, which were concerned with comparing more sophisticated linear reference alignment strategies with graph-based alignment strategies, the new methods 'MajorFlow', 'RandFlow' and 'RandFlow-LD' outperform 'vg' in terms of compute resource requirements (quite substantially), while 'vg' keeps (small, but still marked) advantages in terms of the quality of the alignments computed. While MajorFlow does not lead to increased sensitivity when aligning reads, and suffers in particular when dealing with the (the most diverse) African super population, RandFlow and RandFlow-LD are rivaling 'vg' in this respect. Still, RandFlow and RandFlow-LD suffer from (certainly significantly) more allele bias relative to the reads they are able to align than 'vg'.

I found the study interesting to a certain degree: it puts a particular focus on how one can integrate the 1KG super populations when trying to profit from resource friendly linear reference alignment technology.

However, it also remained unclear to me to what degree super populations really can enhance linear alignment strategies, because the results do not necessarily look uncommon to me in comparison to alternative linear reference based alignment strategies. For example, it seems to become (ever more) obvious that when dealing with reads from genomes sufficiently close to the common reference genome (like reads from non-African super populations), linear reference based strategies may already be a sufficiently satisfying option, which is not necessarily surprising.

Overall, the study left me behind with the following substantial questions:

* Why should randomly picking alleles, instead of using major alleles lead to advantages? Couldn't the randomly composed genomes just reflect statistical outliers? The authors should repeat the procedure of randomly picking alleles several times, also because the amount of reads used is rather small, and refers to only one of the chromosomes. =

=response begin==

We agree that it would put the method on more solid ground if we could measure the variability induced by the random choices of allele. In particular, we thought about how we could do this in a way that would allow us to compare this variability against other sources of variability, such as between random donor individuals and between tools.

To achieve this, we built 15 sets of RandFlow-LD chr21 references using different random seeds, which in turn induced different random allelic choices. We then re-ran the chr21 simulation experiment for each. As we did in Figure 2, we ran alignments using these 15 RandFlow-LD references on 100 samples for alignment sensitivity and 25 samples for allelic bias metrics. We now describe these new results in Note S2, Figure S10-12. We further compared the results with vg’s. To quote the caption of Figure S10, "The heights of the boxes convey variability due to the random seed, whereas the vertical spread of same-color boxes conveys variability due to genetic differences between donor individuals. Notably, variability due to random seed is substantially lower compared to variability due to donor-individual genetics." Note that our boxplots include all outliers.

We would point out that our simulation experiments are quite extensive. For the alignment sensitivity experiment (Figure 2a), we simulated 2M reads for each of the 100 samples, 200M reads in total; while the vg and HISAT2 studies both simulated 10M reads for similar experiments (vg: Figure 2; HISAT2: Figure 4). For the allelic bias experiment (Figure 2bc), we simulated 20M reads for each of the 25 samples, totaling 500M reads. Our experiments spanned five 1000 Genomes super populations. Beyond these, we further studied real whole-human sequencing data, inclusive of 1.4 billion reads. While the variability experiments were performed only for the chr21 simulation and not for the full genome, this was because repeated experiments on the whole-human dataset would not have been doable in the time available. ==response end==

* How would 'vg' behave, if the exact same alleles that were picked for the generation of the linear references used here, were inserted into the variation graphs? The way experiments are designed does not reflect a true head-to-head comparison. Of course, however, the way 'vg' is used is perfectly fine and reflects a standard strategy. To my understanding, choosing 6 genomes and aligning against them linearly, by using linear alignment tools, reflects to consider a variation type graph that hosts the variants incorporated into the 6 genomes and specifying the paths that reflect the 6 genomes along with the graph structure when aligning against the graph.

==response begin==

We agree that the work is strengthened by adding a "head-to-head" comparison. As suggested, we compiled the set of variants used by the RandFlow-LD references, containing the union of the major alleles (used for first-pass alignments) and the variants used in five superpop references (used for second-pass). We built a vg graph with this variant set and evaluated its performance on chr21, setting up the experiments in the same way as the original Section 2.2. We showed that using RandFlow-LD achieved both lower bias and higher alignment sensitivity, compared to vg using the RandFlow-LD variants. We also evaluated different vg settings including using a linear genome, an Allele Frequency (AF) >=1% graph, the RandFlow-LD graph, and an AF>=10% graph.

All of these new results are discussed in the new results Section 2.6. We compared the allele frequency distribution between the RandFlow-LD variant set with the AF>=10% set in Note S2 and Table S5. Results using the vg-RandFlow-LD graph and other graph-based methods are shown in Figures S6-8. ==response end==

* 'RandFlow(-LD)' can be interpreted as using the major allele reference, together with five more genomes, each of which sampled from alleles characterizing one of the 5 super populations.

What I would find a truly interesting idea is to not only pick 5 randomly (and by population constraints consistently) sampled genomes, but increase the amount of randomly sampled such genomes. One would then further explore the limits of such a strategy: at which number of (by population consistently picked) random genomes would advantages in terms of compute resource expenses disappear (in comparison to 'vg'), and, at the same time, would one possibly truly rival the performance of 'vg' in terms of alignment quality/biases?

In other words, this could shed light what the trade-off between compute resource requirements on the one hand and alignment quality on the other hand looks like when trying to maximally increase alignment quality, while adding randomly sampled genomes, one by one. In other words: how does alignment quality behave plotted against increasing costs in terms of resources.

Possibly, the linear alignment strategy is better behaved with respect to the balance of resource costs and alignment performance.

==response begin==

We agree such an experiment would elucidate the trade off between accuracy and computation. We performed an additional set of experiments wherein we generated a RandFlow-LD reference for each of the 26 1000 Genomes Project populations. This is substantially more than in our other experiments, where we used just the 5 higher-level superpopulations. We called this RandFlow-LD-26 and we showed that it improved alignment accuracy and outperformed vg (with a AF>=10% graph) on all measures of reference bias in both the Chromosome-21 simulation experiment and the wholegenome experiment using real data. We evaluated the computational performance in Section 2.5 and Table 3, showing that RandFlow-LD-26 was still ~2x faster than vg. Overall this reflects -- as we would expect -- that increasing the number of reference genomes used in the second pass yields greater accuracy.

While we could generate still more reference genomes, we stopped at 26 chiefly because we could not think of a good way to generate additional individuals apart from simply re-sampling from the same population or superpopulation distributions. We expect this would lead to diminishing returns, but at "full cost" with respect to computational performance. We have left further exploration of this trade-off for future studies. ==response end==

* While results are concerned with sensitivity, they are never concerned with false positives, i.e. the amount of incorrectly placed alignments. It would be interesting to understand how many not correctly placed alignments were indeed incorrectly placed (and hence are false positives) in comparison to reads that remained entirely unaligned. One could consider incorrect placement worse than refusing to align reads at all, because suggesting incorrect placements could lead to flawed conclusions in downstream analyses.

==response begin==

We agree that including the number of false positives strengthens our alignment accuracy comparisons. We added new Figures S14 and S16 to show the number of unaligned reads of the simulated chr21 and the real whole-genome experiments respectively. We also provided the number of incorrect alignment for the simulated experiment in new Figure S15. These results show that the reference flow methods reduced the number of unaligned reads in both simulated and real data experiments, and reduced the number of incorrect alignments in simulated experiments. We included the discussion in Section 2.3. ==response end==

There are some further major concerns:

* The entire paper reads like a case study. There are not necessarily any truly novel methodical achievements other than an alignment Protocol.

==response begin==

We understand the reviewer's point of view, but we see it a different way. The manuscript presents an entirely new framework (reference flow) together with specific, new, highly practical methods within that framework. The RandFlow methods are new and have not been applied in any prior study. They are unique in their ability to avoid the same amount of reference bias as graph aligners, but at a fraction of the computational cost. While some components of the framework use existing tools (e.g. Bowtie 2), the framework itself could not have been made from off-the-shelf software. It required new software for generating the representative genomes, for merging alignments, for lifting alignments to the destination reference genome, and for coordinating the entire process of committing and deferring alignments. While we could have achieved more novelty by implementing another new approach for aligning to graphs, we think we have done something more profound by proposing a truly practical framework into which either linear or graph aligners (or both) can be fit.

Further, the question of what genomes to include in the second pass required the scientific investigation presented here. The result we saw (random individuals outperforming major-allele references) was neither what we expected, nor what recent literature (e.g. Ballouz et al) was suggesting. ==response end==

* One of the major advantages of 'vg' is to reflect a coordinate system that applies for several genomes together. How much time does one loose in the methods presented here for unifying alignments?

==response begin== This is a good point; "lifting" alignments to the final coordinate system is not computationally trivial. Further, our approach must also merge alignments from all the previous steps and select the best alignment for each read. In our experiments, the computational cost of lifting and merging results are included in our measurement in Section 2.5 and Table 3. Compared to the overall runtime of RandFlow-LD, lifting takes 7% and merging the results takes 1%. While it is not trivial, it is also not the bottleneck. ==response end==

* RandFlow and RandFlow-LD require to generate genomes by random principles. The generation of the respective genomes also costs time. To what degree does the generation of these genomes play a role when considering runtime? Or is this just completely negligible? Possibly also fixing the sampled genomes once and forever; but then how can one make sure that the random genomes do not reflect outliers that lead to bad alignment quality on at least some of the chromosomes?

==response begin==

Generating a set of RandFlow or RandFlow-LD FASTA files takes 13 hours and 24GB memory using a 5-threaded Python script. Indexing a single human reference takes 3- -4 hours using 8 threads on a single computer. We consider this cost to be modest for two reasons. First, the cost is small compared to the cost of building graph indexes for vg. Construction of a vg graph and index for human typically uses over 100 GB of memory. In our hands, the process is slow and can fail for large genomes and sets of variants. HISAT2 has similar drawbacks; it is vulnerable to exponential blowup in the indexing step when many unphased variants are near each other.

Another point about cost is that it can be "amortized" over many runs of the framework. As is typical in read alignment tools, we can consider the cost of generating and indexing the genomes as being incurred in an "offline" phase; references need to be generated and indexed only once. Users can simply download these and reuse them across many sequencing data analyses.

We addressed the concern about the “outlier” genome in our first response to this reviewer. ==response end==

MINOR:

* Citation 10 (which I thoroughly read earlier, and not to my surprise, still is not published) -- to the best of my understanding -- does not justify by itself to pursue reference based approaches, instead of graph based approaches. Many of the analyses seem to be flawed because of the small amounts of data used. In my opinion, the baseline method seems to be rather contrived and to my judgment is not necessarily a *baseline* method. The advantages on reference reads when comparing with graph based approaches cannot be considered an advantage when trying to overcome issues when trying to align reads that harbor non-reference variation. So I would possibly use additional citations in the hunt for evidence that linear reference based alignment strategies could have advantages. (see https://genomebiology.biomedcentral.com/articles/10.1186/s13059-020-01963-y for example)

==response begin==

We agree and we have cited the CHOP paper at the end of the second paragraph of the Discussion section. We added a sentence to the end of that paragraph reading: "Our results also reinforce that a linear aligner can be extended to incorporate variants and exhibit similar accuracy to a graph aligner." ==response end==

Intro: - p2, l29: why 'rough' upper bound? Isn't this the ideal scenario for a linear reference aligner, so just the 'ultimate' upper bound? (am I overlooking something here?)

==response begin==

This is a good question. While it is true that the personalized reference is ideal for a linear aligner, the accuracy of alignment results are also affected by alignment heuristics. We added the following explanation in section 2.2, just after introducing the idea of the rough upper bound:

--

We call this a "rough" upper bound because, while the personalized reference is ideal in that it contains the correct variants, the accuracy of alignment is also affected by tool-specific heuristics. A true upper bound would be hard to obtain, so we settle for the rough upper bound provided by the personalized genome, as in previous work [Citing FORGe and the recent bovine pan genome paper: 10.1186/s13059-020-02105- 0]. -- ==response end==

Discussion:

- p14, l16: "In principle are not difficult to include..." (broken sentence)

==response begin==

Thank you; we fixed this ("In principle they are not difficult to include"...) ==response end==

Reviewer #3:

My overall impression is that this is a good study, the method is a reasonable tradeoff in time and memory compared to state of the art, vg. However the results of the experiment show minor improvements in sensitivity, and the ref to alt ratio goes from 1.5% bias down to 0.5% bias.

The results as presented are a bit underwhelming almost raising the question of "why bother". What I think needs to be communicated properly is that the average is hiding more serious artefacts that can cause trouble in downstream analysis and interpretation. To do this I would recommend working with a specific example in the introduction, e.g. from RNA-seq or CHIP-seq (e.g. like this paper https://genomebiology.biomedcentral.com/articles/10.1186/s13059-020-02038-8), where a misalignment bias can flip a result. If the focus is supposed to be on sequencing of DNA the next obvious step is variant calling, how are these sites affected, e.g. by running GATK on them?

I would also recommend running this on real data from GIAB which is our best approximation for a gold standard

==response begin==

We agree that the argument could be improved by investigating which specific parts of the genome are benefiting most from the reduced reference bias. We added a new analysis of where the "strongly biased" HET sites from the whole-genome experiment (using real data) fall with respect to RepeatMasker annotations. We describe this result in a new paragraph at the end of section 2.4. We showed that reference flow methods reduced the number of strongly biased HET sites in common repeats such as L1 and Alu compared to GRCh38. For example, when using RandFlow-LD-26, the number of strongly biased sites in L1 was reduced from 10,288 to 3,560, a 65% reduction.

A strength of this study is that it measures reference bias directly, using measures like REF-to-ALT ratio and number of highly biased sites. We can be confident that reducing these measurements should provide downstream tools, such as variant callers, with better data for making calls. Further, if this did not turn out to be the case (less-biased alignments leading to worse downstream calls), we would not know whether to blame the upstream or downstream methods. Overall, we think the clearest argument comes from measuring reference bias changes directly, as done here. ==response end==

Comments

page 2, line 13 not clear whether misalignment or unalignment, also mapping vs alignment

==response begin== Thank you for pointing this out. We replace the sentence with “we can achieve higher alignment sensitivity” (“sensitivity” replaces “accuracy”). ==response end==

page 3, line 12 Not clear which reads, "all reads" are, only later becomes clear they are simulations

==response begin== The opening paragraph in Section 2.2 describes the alignment strategy taken. In this context "all reads" refers generically to the input reads, whether from a simulator or otherwise. It's in the following paragraph that we begin to describe the simulation experiment, and this is where we describe the reads as coming from a simulation. We think the current phrasing conveys this well. ==response end==

page 3, figure 1,a,b Matched is missing from b,c, no explanation in legend Cross between vg and Randflow-LD, hard to compare, keep the order Sensitivity ranges from 0.9275 to 0.9285, this is 1000 reads out of 1M.

==response begin== Thank you for the suggestion. We modified the figure (new Figure 2) to keep them in order. ==response end==

page 6, lines 33-40 Use of real data is good, but can be improved by using genome in a bottle on the same sample, trios for NA12878 and a "truth" set of variants are available, while not as good as a simulation you get real datasets. Also, GIAB, has a high confidence region defined, covers 70% of the genome, in which short reads are "easy", it would be interesting to see the performance of the tools on this regions vs outside.

==response begin== We agree, and we extended our bias evaluation for the real data experiment to include stratifications of the GIAB v3.3.2 confidence level. We still use the 1000-Genome Project callset as “truth” because that callset provides calls outside of high-confidence regions, and so do not exclude clinically important genes (https://genomemedicine.biomedcentral.com/articles/10.1186/s13073-016-0269-0). We expect the community will be interested in bias as measured in both low- and highconfidence regions. ==response end==

page 6, line 46 basied, typo

==response begin== We corrected the typo, thank you. ==response end==

page 8, line 44 8% of the CPU It is not clear at this point (or in the paper) that vg was only run on the same reads as the majorflow, this should be laid out clearly at the start. What is the effect of running vg on the entire read set, how close is it to the personalized alignments?

==response begin== We aligned all reads using vg in that experiment. We clarified this in Section 2.3, where we now say:

-- We built a vg index using chromosome 21 of the GRCh38 primary assembly as the base, and including all variants from the 1000-Genomes GRCh38 callset having allele frequency at least 1% and aligned all reads to the graph. -- ==response end==

page 10, line 24 for 0.2-0.8, Given an average coverage of 30x and assuming a Poisson coverage, what is the probability of observing this by chance given a perfectly unbiased sample.

==response begin== We performed the following simulation to estimate the expected number of strongly biased sites if reads for each haplotype were drawn from a Poisson distribution with rate parameters set to half the observed average coverage (46). As the number of draws, we used the number of HET sites (2,068,262). The simulation R code is as follows:

```

> nsites <- 2068262

> cov <- 46.0

> as <- rpois(nsites, cov/2)

> a_bal <- as / (as + rpois(nsites, cov/2))

> sum(a_bal <= 0.2 | a_bal >= 0.8) [1] 190

```

Thus, the expected number of strongly biased sites was less than 200, orders of magnitude lower than the observed numbers of strongly biased sites. This is perhaps not surprising considering the many reasons why there is bias in practice, some related to reference bias, others relating to non-Poisson variations in coverage due e.g. to PCR and fragmentation. ==response end==

I would recommend adding a table, supplementary or in the paper, with the following statistics

What fraction of the simulated reads were unaligned and which were poorly aligned.

For each method How many of the poorly aligned reads were mapped to different regions with your method.

How many of the unaligned were mapped to a unique region in the genome

==response begin== We agree; we added Figures S14 and S16 to show the number of unaligned reads for the simulated and real experiments respectively and Figure S15 to show the number of incorrect alignments in the simulated experiment. ==response end==

**Second round of review**

**Reviewer 1**

The authors have thoroughly addressed all of my concerns and added a substantial number of new experiments.  I am satisfied with the current manuscript and have no remaining questions or concerns.

**Reviewer 2**

I am quite impressed by the additional analyses the authors carried out.

Just a philosophical discussion, but ...

I still tend to disagree on whether the authors provide a new method. I think the authors describe a protocol by which one can optimally exploit the particular set of genomes in terms of linear alignment methods. It remains rather unclear to me, whether the method is of more general value than just that (which however may have great practical value, of course!).

For example, could I possibly apply the protocol also to other than human genomes? I am also missing a bit more of an explanation why picking random alleles for crafting the linear genomes against which to align has such a powerful effect? It still feels a bit like serendipity to me. (it certainly isn't! -- but what's the reason?, and when understanding this, can I make use of the underlying principle in other settings, too?)

For a method, I'd expect a well-motivated methodical principle that one can apply in more generality.

Note that vg can be applied to any set of sufficiently (evolutionarily) coherent genomes, once turned into a graph.

Can you make such a rather general claim for RandFlow as well? Or do I have to resort to vg when the particular conditions (human-like genomes carrying randomly picked alleles from superpops) described in your paper do not apply?

**Reviewer 3**

The authors have answered all of the points raised in my review. I think the results are strengthened with the addition of the GIAB dataset.

Minor notes:
In plots the y-axis is often cut off, with no origin.

In many cases, e.g. figures like Fig2 and S15 the y-axis on the RHS provides a natural origin, perhaps it should be moved to the left as the grids in ggplot do not align with it.

For S16, the plot should include the origin.

**Authors Response**

**Point-by-point responses to the reviewers’ comments:**

Reviewer reports: Reviewer #1: All of my previous comments have been well-addressed, and I have no further comments for the authors.

== begin response == We thank the reviewer for their helpful comments. == end response ==

Reviewer #2: Just a philosophical discussion, but ... I still tend to disagree on whether the authors provide a new method. I think the authors describe a protocol by which one can optimally exploit the particular set of genomes in terms of linear alignment methods. It remains rather unclear to me, whether the method is of more general value than just that (which however may have great practical value, of course!).

For example, could I possibly apply the protocol also to other than human genomes? I am also missing a bit more of an explanation why picking random alleles for crafting the linear genomes against which to align has such a powerful effect? It still feels a bit like serendipity to me. (it certainly isn't! -- but what's the reason?, and when understanding this, can I make use of the underlying principle in other settings, too?)

For a method, I'd expect a well-motivated methodical principle that one can apply in more generality. Note that vg can be applied to any set of sufficiently (evolutionarily) coherent genomes, once turned into a graph. Can you make such a rather general claim for RandFlow as well? Or do I have to resort to vg when the particular conditions (human-like genomes carrying randomly picked alleles from superpops) described in your paper do not apply?

== begin response ==

We think the nature and value of the new Reference Flow method is described well in the current manuscript. Reference Flow can indeed be applied to any set of relevant genomes. Further, Reference Flow does not require that the genomes be placed into an overall graph -- i.e. global coordinate system -- and only requires that the secondpass genomes each be aligned to the target reference. All in all, what we describe here is just as coherent, general, and "method-like" as vg, while being far more computationally efficient and practical. == end response ==

Reviewer #3: Minor notes: In plots the y-axis is often cut off, with no origin.

In many cases, e.g. figures like Fig2 and S15 the y-axis on the RHS provides a natural origin, perhaps it should be moved to the left as the grids in ggplot do not align with it.

== begin response ==

In all the cases where the original left-hand vertical axis did not have a logical origin, we switched the y axes so that the left-hand axis (which governs the gridlines) used the gain-of-personalization scale, which does have a logical origin. We did this for Figure 2a, Figure S1, S3, S12. == end response ==

For S16, the plot should include the origin.

== begin response == We included the origin in Figure S8 (previous S16). == end response ==
